# ST Elevations in the Era of COVID-19

**DOI:** 10.1155/2020/8845627

**Published:** 2020-08-06

**Authors:** Charlie Joseph Sang, Brittain Heindl, Gregory Von Mering, Brigitta Brott, Robert S. Kopf, Indranee Rajapreyar

**Affiliations:** ^1^Internal Medicine—Pediatrics Residency Program, Department of Pediatrics, University of Alabama at Birmingham, Birmingham, AL, USA; ^2^Division of Cardiovascular Disease, Department of Medicine, University of Alabama at Birmingham, Birmingham, AL, USA; ^3^Section of Interventional Cardiology, Division of Cardiovascular Disease, Department of Medicine, University of Alabama at Birmingham, Birmingham, AL, USA; ^4^Division of Pulmonary, Allergy, And Critical Care Medicine, Department of Medicine, University of Alabama at Birmingham, Birmingham, AL, USA; ^5^Section of Heart Failure and Transplantation Cardiology, Division of Cardiovascular Disease, Department of Medicine, University of Alabama at Birmingham, Birmingham, AL, USA

## Abstract

Myocardial injury, represented by elevated cardiac enzymes, has been associated with increased morbidity and mortality in severe acute respiratory syndrome coronavirus-2 (SARS-CoV-2) infections. Coronavirus disease 2019 (COVID-19) has created unique challenges in approaching patients with acute ST-segment changes. We describe two distinct cases of ST elevation on electrocardiogram occurring in patients with COVID-19 and review important diagnostic and management considerations for the front-line clinician.

## 1. Introduction

In December 2019, the novel severe acute respiratory syndrome coronavirus-2 (SARS-CoV-2) emerged in Wuhan, China. Originally characterized by its diversity of respiratory presentations [1], it is now known to have wide-ranging effects on the cardiovascular system including venous and arterial thromboembolism, coronary plaque instability and rupture, and direct myocardial, pericardial, or epicardial damage. Electrocardiographic abnormalities are prevalent in patients with COVID-19 and are associated with a worse prognosis [2]. In a cohort study published by Shi and colleagues, electrocardiograms (ECGs) were universally abnormal in the setting of elevated biomarkers, suggesting some component of intrinsic myocardial (and potentially epicardial) damage [3]. We describe two cases of ST elevation on ECG associated with COVID-19 and highlight diagnostic and management principles that have become consensus at our institution.

## 2. Case 1 Presentation

A 38-year-old male with hypertension presented following a motor vehicle collision with traumatic injuries. Physical exam on arrival revealed temperature of 36.6°C, tachycardia (154 bpm), blood pressure 142/69 mmHg, and saturating at 93% on a nonrebreather. Breath sounds were decreased on the left side. Initial labs revealed a lactic acid of 3.0 mMol/L (ref. 0.5–2.2), acute kidney injury with serum creatinine 1.5 mg/dL (ref. 0.7–1.3), high sensitivity Troponin-I (hs-Troponin-I) of 1295 ng/L (ref. 3–20), WBC of 7.43 10^3^/cmm (ref. 4.0–11.0), PTT 27 s (ref. 25–35 s), and INR 1.05. Chest X-ray revealed elevated left hemidiaphragm, left hemopneumothorax, complete opacification of the left lung, and rightward mediastinal shift. He was taken to the operating room emergently for surgical repair. Initial ECG showed sinus tachycardia. Following surgery, he remained intubated for acute hypoxic respiratory failure.

**Figure 1:**
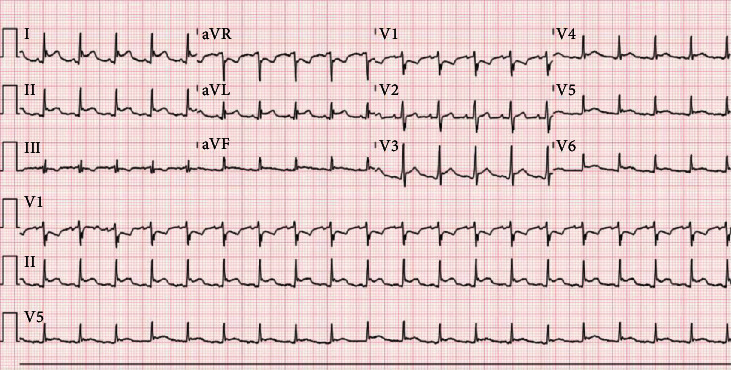
Electrocardiogram with tall ST-segment elevations in leads V3-V6, I, II, and aVL with ST-segment depressions in V1, aVR, and III.

**Figure 2:**
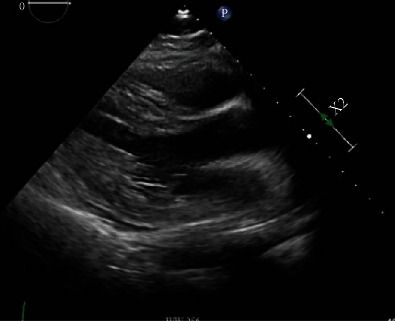
Transthoracic echocardiogram showed a small left ventricular cavity, thick left ventricular walls, and trivial pericardial effusion.

Two days later, he developed fever to 100.2°F. Recently, patients in the intensive care unit had been diagnosed with nosocomial COVID-19; therefore, he was tested and his SARS-CoV-2 RT-PCR was positive. A viral respiratory panel returned positive for adenovirus. Given the time-sensitive nature of a peritrochanteric femoral fracture repair, the operation was performed with appropriate precautions. Following surgery, he had ST-segment elevations on telemetry. ECG confirmed tall ST-segment elevations in leads V3-V6, I, II, and aVL with ST-segment depressions in V1, aVR, and III (Figure 1). A bedside echocardiogram showed normal left ventricular ejection fraction, normal wall motion, thick left ventricular walls, and a small left ventricular cavity. Hs-Troponin I returned at 108 ng/L, and two hours later, was at 106 ng/L. Based on the clinical presentation, a diagnosis of myopericarditis was given. No COVID-19-specific therapies were recommended. Formal echocardiogram was obtained the following morning, confirming earlier findings (Figure 2).

**Figure 3:**
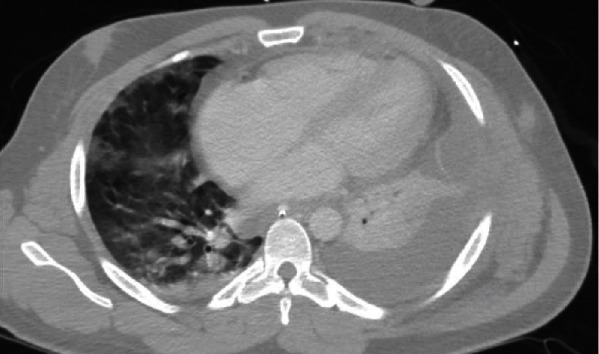
CT chest shows left-sided pleural effusion with loculations and atelectasis. Nodular groundglass opacities are present in the right upper and middle lobes. There is a demonstration of a small pericardial effusion.

Over the following three days, he developed progressive hypoxemia despite optimal ventilator settings. A repeat CT of the chest was obtained revealing increasing size of a left-sided pleural effusion and interval development of nodular, ground glass opacities in the right upper and middle lobes (Figure 3). Given the increasing size of his pleural effusion, a chest-tube was reinserted for decompression, and a bronchoalveolar lavage was performed to assess for coinfection, revealing pan-sensitive *E. coli*. Following appropriate treatment with antibiotics, he was extubated on hospital day 10. He was discharged to an inpatient rehabilitation unit on hospital day 16. At one-month follow-up, he was doing well and denied chest pain or dyspnea on exertion.

## 3. Case 1 Discussion

This patient tested positive for COVID-19 within 3 days of admission for severe trauma. Upon completing surgery, his ECG demonstrated diffuse ST-segment elevations concerning for acute coronary syndrome versus myopericarditis. COVID-19 is associated with ECG findings of myocardial ischemia with subsequent catheterization without significant coronary stenosis [4]. Myocarditis and pericarditis are known complications in patients with COVID-19 [5]. Bedside echocardiogram was key to distinguishing ST elevation myocardial infarction from myopericarditis. The absence of wall motion abnormalities, normal left ventricular function, presence of myocardial wall thickening, and pericardial effusion made myopericarditis more likely. In patients with COVID-19, risk of exposure in the catheterization lab must be weighed against those of delayed revascularization. While coronary CT angiography could be considered in this scenario, we felt it would be of little yield. In trials comparing the use of early coronary CT angiography in patients with low-intermediate likelihood of acute coronary syndrome in the emergency setting, there was no difference in the rate of major adverse cardiac events when compared to a traditional care model [6]. Our patient had stable troponins and higher likelihood of myopericarditis. Given these findings, coronary CT angiography would have caused increased exposure to radiation, created additional viral exposure to radiology technicians, contaminated imaging materials thus delaying access to other patients, and had limited data that it would reduce major adverse cardiac events in this setting.

While his myopericarditis was attributed to COVID-19, we must also consider the possible effects of adenovirus, as this virus is also known to cause myopericarditis [7]. While the true etiology cannot be determined in the absence of endomyocardial biopsy with PCR sampling, we speculate COVID-19 had a predominate role given the timing of his presentation and radiographic features.

Optimal treatment for COVID-19 myopericarditis is unknown. In the absence of significant ventricular dysfunction, treatment of myopericarditis is similar to treatment of acute pericarditis [7]. In contrast to pericarditis, the use of nonsteroidal anti-inflammatory drugs should be used with caution, as these agents may enhance inflammatory processes and increase mortality [7]. Colchicine has shown benefits in both the acute and recurrent forms of pericarditis; however, there is limited data on its use in myopericarditis, and further studies are needed [8]. A trial in Montreal is currently evaluating the effect of colchicine to determine if it reduces the severity of illness in COVID-19 [Colchicine Coronavirus SARS-CoV2 Trial (COLCORONA) (COVID-19)]. Corticosteroids are associated with significant recurrence rates for myopericarditis, perhaps due to effects on viral replication and resultant aggravation of pericardial involvement [8, 9]. Given these findings, we recommend against the use of systemic corticosteroids in the setting of viral myopericarditis. Overall, gaps remain in the literature regarding treatment for myopericarditis, especially with regards to COVID-19, and treatment remains largely supportive. Myopericarditis generally carries a good prognosis [10]. In a multicenter, prospective cohort study, 486 patients with either pericarditis, myopericarditis, or perimyocarditis were followed for a median of 36 months. In this cohort, LV function on initial evaluation was normal or nearly normal in each patient, with recovery of LV function occurring in >90% in patients with myopericarditis [10]. Although our patient has not yet had a follow-up echocardiogram, we believe he will continue to improve without serious complications.

## 4. Case 2 Presentation

**Figure 4:**
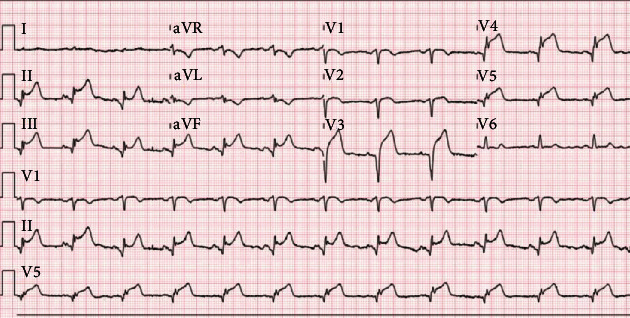
Electrocardiogram exhibiting ST-segment elevations in both the anterolateral and inferior leads.

**Figure 5:**
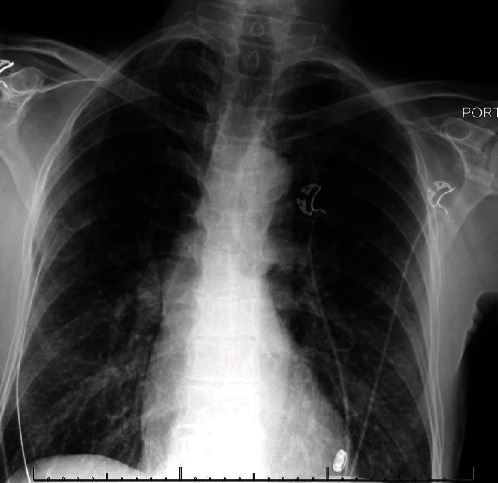
Chest X-ray revealing normal cardiac silhouette size. Mild interstitial edema bilaterally suggestive of CHF. No large effusion or dense consolidation.

**Figure 6:**
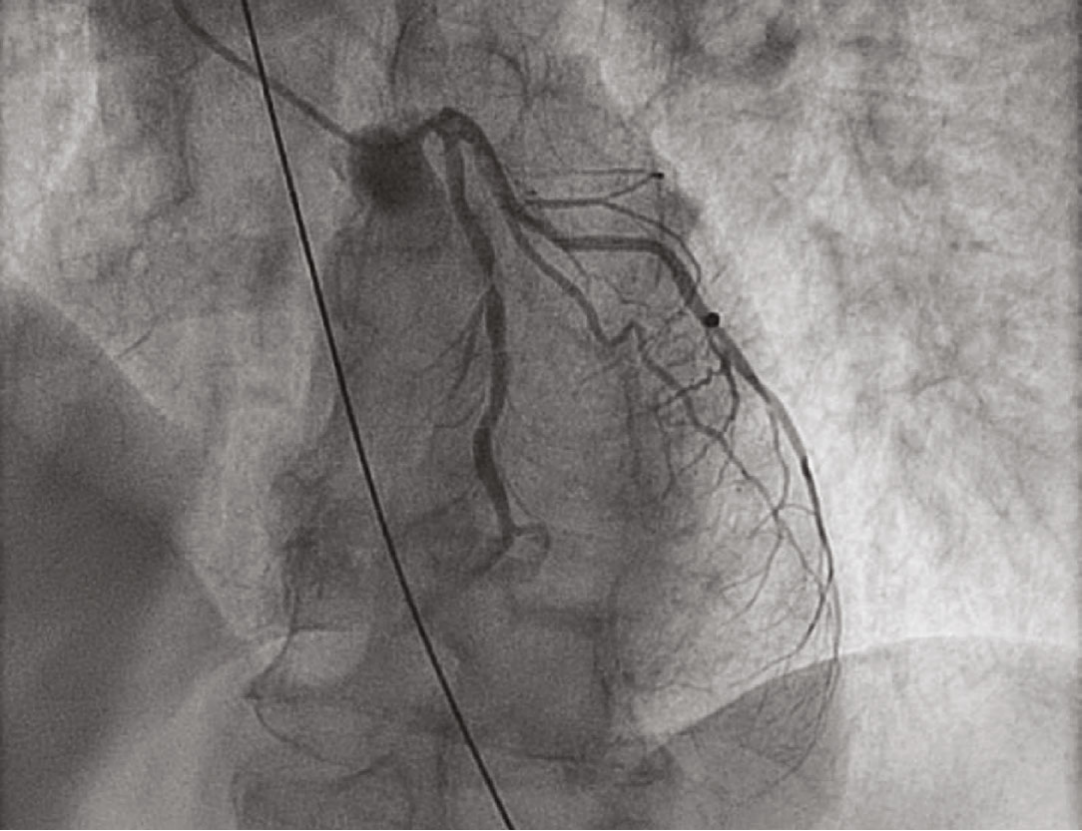
Coronary angiogram revealing mid-LAD stenosis.

A 68-year-old male with chronic obstructive pulmonary disease and tobacco dependence presented to the emergency department with nausea, chest pain, and shortness of breath. He endorsed progressive cough over the preceding two weeks. On examination, his HR was 72 bpm, blood pressure 115/91 mmHg, respiratory rate 22, and saturating 98% on 2L nasal canula. He appeared in significant discomfort. He had a normal rate, regular rhythm, and no murmurs. The lungs were clear to auscultation. There was no peripheral edema. ECG revealed ST-segment elevations in both the anterolateral and inferior leads (Figure 4). Chest X-ray revealed a normal cardiac silhouette, with bilateral interstitial opacities felt to represent pulmonary edema (Figure 5). The catheterization lab was activated, and aspirin, ticagrelor, and heparin were administered. Given the history of cough, a SARS-CoV-2 RT-PCR was obtained before he was taken to the catheterization lab. The patient was taken to a designated COVID-19 catheterization lab with infection control precautions. Coronary angiography revealed severe stenosis of the mid-left anterior descending artery (Figure 6). Mild and nonobstructive disease was present in the other two vessels. Two drug-eluting stents were placed in the left anterior descending artery with restoration of flow. He was transferred to the Cardiac Care Unit with isolation precautions. Initial labs revealed hs-troponin I 27 ng/L, brain natriuretic peptide 101 pg/mL (ref. 0.0–100.0), PTT 29 s, INR 0.94, and WBC 8.5 10^3^/cmm. Echocardiogram obtained the following morning revealed a left ventricular ejection fraction of 20-25%. The mid and distal segments of the inferior, inferoseptal, anterior, and anteroseptal left ventricular walls were severely hypokinetic, consistent with acute myocardial infarction (Video1). Thirteen hours later, hs-troponin I peaked at 159,040 ng/L. The following day, his SARS-CoV-2 RT-PCR reported positive. Following his percutaneous intervention, the patient did well. He was discharged home on hospital day 3 with goal-directed therapy consisting of aspirin, atorvastatin, lisinopril, metoprolol, and ticagrelor.

**Figure 7:**
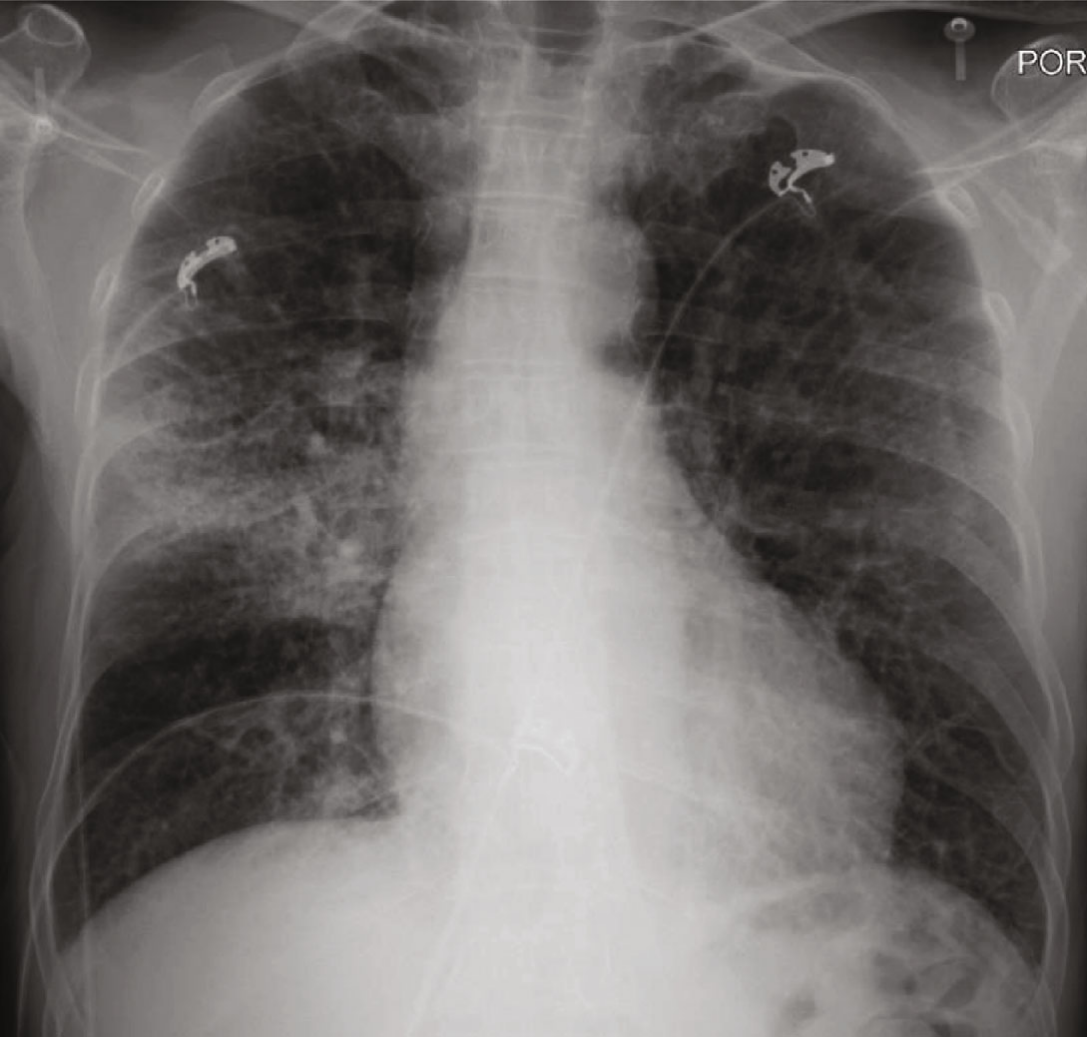
Chest X-ray showing interval development of right middle lobe airspace opacity concerning for pneumonia. There are diffusely increased interstitial markings, which may be seen with mild pulmonary edema.

Three days later, he presented with acute hypoxemic respiratory failure requiring mechanical ventilation. Chest X-ray revealed interval development of a right middle lobe airspace opacity consistent with pneumonia (Figure 7). ECG prior to intubation revealed inferior and anterolateral ST elevations similar to his prior ECGs. The dynamic inferior ST-segment changes were felt to be related to coexistent COVID-19 infection and recent myocardial infarction. Repeat echocardiogram did not exhibit any changes. Given his down trending hs-troponin-I (9,782 ng/L), it was felt that this presentation was inconsistent with reinfarction. He was admitted to the intensive care unit and started on broad-spectrum antibiotics. Given his recent diagnosis of COVID-19, associated inflammatory markers were obtained and revealed CRP 70.86 mg/L (ref. 0.00–10.90), ESR 17 mm/hr (ref. 0–10), lactate dehydrogenase 863 units/L (ref. 120–240), Ferritin 221 ng/mL (ref. 23.9–336.2), D-dimer 946 ng/mL (ref. 0–240), interleukin 6 of 5 pg/mL (ref. ≤5), and procalcitonin 0.02 ng/mL (ref. 0.00–0.07). He was considered for enrollment in the Remdesivir trial; however, repeat testing for SARS-CoV-2 was negative. He was extubated two days later and on hospital day 4 was discharged on levofloxacin to complete a 7-day total course for pneumonia.

At his three-month follow-up, he endorsed feeling well and denied functional impairment. Repeat ECG showed sinus bradycardia and nonspecific ST-T wave changes, and echocardiogram (Video2) revealed a persistently depressed EF of 25-30% with severe left ventricular dysfunction. He was referred for ICD placement with plans for follow-up in 2 months.

## 5. Case 2 Discussion

Patient 2 experienced a presentation classic for acute ST-segment elevation myocardial infarction. The degree to which COVID-19 contributed to his coronary event is worth considering. The patient had hypertension, dyslipidemia, and tobacco dependence—and thus, he was at risk for coronary atherosclerosis. Viral illnesses, especially influenza, are recognized to contribute to coronary events [11]. The etiology of this association is the increased inflammatory response predisposing at-risk patients to plaque rupture and subsequent thrombosis [11].

This scenario also raises important questions in the management of COVID-19 patients with ST elevation myocardial infarction in the United States, where percutaneous coronary intervention (PCI) is the standard of care. Institutions in China have developed alternate care pathways involving rapid molecular testing and predominance of systemic fibrinolytic therapy [12]. These pathways are controversial in the United States for which fibrinolytic therapy is not routinely recommended in PCI-capable centers [13]. While many concerns have been raised regarding appropriate precautions for coronary angiography in the COVID era, this case shows successful activation and execution of the catheterization lab in a patient with suspected, and later, confirmed COVID-19. Diverging slightly from standard protocol, there was pause to discuss the patient's risk of having COVID-19. The patient's history of cough was enough to warrant testing and institute precautions in the catheterization lab. Despite this delay, the angioplasty balloon was expanded within the 90-minute recommended window. The success of this execution is, in part, due to maintaining a catheterization lab dedicated to “persons under investigation” and COVID-19-positive patients.

In patients with risk factors for atherosclerotic coronary disease, the utility of “cardioprotective” medications in preventing COVID-19-induced myocardial infarction has been controversial. As SARS-CoV-2 utilizes the angiotensin-converting enzyme 2 for infecting the host cell, debate exists regarding the safety of angiotensin-converting enzyme inhibitors (ACEi), angiotensin receptor blockers (ARB), and mineralocorticoid antagonists, as these medications are known to upregulate angiotensin-converting enzyme 2 [14]. Statin therapy is also known to play a role in upregulation of angiotensin-converting enzyme 2, which may be partially responsible for the cardiovascular protection elicited with this medication class [14]. In a retrospective case series, there were no significant differences in the severity of disease or mortality in patients taking an ACEi/ARB for hypertension [15]. As such, it is currently recommended to continue ACEi/ARBs in patients diagnosed with COVID-19 infection [16].

The use of ticagrelor in the setting of COVID-19 myocardial infarction requires further attention. As illustrated by Sexton et al., ticagrelor was found to reduce levels of proinflammatory cytokines such as IL-6 and was associated with improved lung function in patients with pneumonia [17]. In addition to these favorable effects, ticagrelor has also been shown to have antimicrobial properties against gram-positive bacteria [18], a frequent complication in patients requiring prolonged mechanical ventilation. Severe disease in COVID-19 is also associated with sepsis-induced coagulopathy and increased mortality [19], and ticagrelor's anti-inflammatory and anti-platelet effects may prove useful in reducing this phenomenon [20]. Future studies are needed to assess this potentially beneficial therapy.

## 6. Conclusions

We present two cases associated with COVID-19 and ST elevations on ECG. Myocardial injury contributes to excess morbidity and mortality in patients affected by COVID-19, likely in part due to the diverse cardiovascular presentations and challenges with diagnosis. With uncertain clinical presentations, the clinician may use echocardiogram to better assess the predictive value of invasive interventions such as angiography and use this tool to guide treatment while reducing the risk of unnecessary exposure in the setting of COVID-19.

## Abbreviations

SARS-CoV-2: Severe acute respiratory syndrome coronavirus-2
COVID-19: Coronavirus disease 2019
hs-Troponin I: High-sensitivity troponin I
RT-PCR: Reverse-transcriptase polymerase chain reaction
ECG: Electrocardiogram
PCI: Percutaneous coronary intervention.

## Supplementary Materials

Supplementary 1
*Video 1*. Transthoracic echocardiogram exhibiting severely reduced left ventricular ejection fraction (20-25%), hypokinesis of the mid- to distal segments of the inferior, inferoseptal, anterior, and anteroseptal walls with apical akinesis.

Supplementary 2
*Video 2*. The left ventricle is normal in size with normal left ventricular wall thickness. Left ventricular systolic function is severely reduced. Ejection fraction = 25-30%. Dynamic heart model LVEF = 29%. The basilar anteroseptum is markedly hypokinetic. There is thinning and scarring of the mid-anteroseptum, anterior, and anterolateral wall segments. The apex is dilated, thinned, and akinetic.

## References

[1] Guan W. J., Ni Z. Y., Hu Y. (2020). Clinical characteristics of coronavirus disease 2019 in China. *New England Journal of Medicine*.

[2] Driggin E., Madhavan M. V., Bikdeli B. (2020). Cardiovascular Considerations for Patients, Health Care Workers, and Health Systems During the COVID-19 Pandemic. *Journal of the American College of Cardiology*.

[3] Shi S., Qin M., Shen B. (2020). Association of cardiac injury with mortality in hospitalized patients with COVID-19 in Wuhan, China. *JAMA Cardiology*.

[4] Bangalore S., Sharma A., Slotwiner A. (2020). ST-segment elevation in patients with Covid-19—a case series. *New England Journal of Medicine*.

[5] Bonow R. O., Fonarow G. C., O’Gara P. T., Yancy C. W. (2020). Association of coronavirus disease 2019 (COVID-19) with myocardial injury and mortality. *JAMA Cardiology*.

[6] Rybicki F. J., Udelson J. E., Peacock W. F. (2016). 2015 ACR/ACC/AHA/AATS/ACEP/ASNC/NASCI/SAEM/SCCT/SCMR/SCPC/SNMMI/STR/STS Appropriate Utilization of Cardiovascular Imaging in Emergency Department Patients With Chest Pain. *Journal of the American College of Cardiology*.

[7] Imazio M., Trinchero R. (2008). Myopericarditis: etiology, management, and prognosis. *International Journal of Cardiology*.

[8] Imazio M., Bobbio M., Cecchi E. (2005). Colchicine in addition to conventional therapy for acute pericarditis. *Circulation*.

[9] Imazio M., Demichelis B., Parrini I. (2005). Management, risk factors, and outcomes in recurrent pericarditis. *The American Journal of Cardiology*.

[10] Imazio M., Brucato A., Barbieri A. (2013). Good prognosis for pericarditis with and without myocardial involvement: results from a multicenter, prospective cohort study. *Circulation*.

[11] Zhou F., Yu T., du R. (2020). Clinical course and risk factors for mortality of adult inpatients with COVID-19 in Wuhan, China: a retrospective cohort study. *Lancet*.

[12] Welt F. G. P., Shah P. B., Aronow H. D. (2020). Catheterization laboratory considerations during the coronavirus (COVID-19) Pandemic. *Journal of the American College of Cardiology*.

[13] O’Gara P. T., Kushner F. G., Ascheim D. D. (2013). 2013 ACCF/AHA guideline for the management of ST-elevation myocardial Infarction. *Circulation*.

[14] South A. M., Diz D., Chappell M. C. (2020). COVID-19, ACE2 and the cardiovascular consequences. *American Journal of Physiology-Heart and Circulatory Physiology*.

[15] Li J., Wang X., Chen J., Zhang H., Deng A. (2020). Association of renin-angiotensin system inhibitors with severity or risk of death in patients with hypertension hospitalized for coronavirus disease 2019 (COVID-19) infection in Wuhan, China. *JAMA Cardiology*.

[16] Messerli F. H., Siontis G. C., Rexhaj E. (2020). COVID-19 and renin angiotensin blockers: current evidence and recommendations. *Circulation*.

[17] Sexton T. R., Zhang G., Macaulay T. E. (2018). Ticagrelor reduces thromboinflammatory markers in patients with pneumonia. *JACC: Basic to Translational Science*.

[18] Lancellotti P., Musumeci L., Jacques N. (2019). Antibacterial activity of ticagrelor in conventional antiplatelet dosages against antibiotic-resistant gram-positive bacteria. *JAMA Cardiology*.

[19] Tang N., Bai H., Chen X., Gong J., Li D., Sun Z. (2020). Anticoagulant treatment is associated with decreased mortality in severe coronavirus disease 2019 patients with coagulopathy. *Journal of Thrombosis and Haemostasis*.

[20] Omarjee L., Meilhac O., Perrot F., Janin A., Mahe G. (2020). Can ticagrelor be used to prevent sepsis-induced coagulopathy in COVID-19?. *Clinical Immunology*.

